# Protective Effects of *Laminaria japonica* Polysaccharide Composite Microcapsules on the Survival of *Lactobacillus plantarum* during Simulated Gastrointestinal Digestion and Heat Treatment

**DOI:** 10.3390/md22070308

**Published:** 2024-06-30

**Authors:** Honghui Guo, Yelin Zhou, Quanling Xie, Hui Chen, Ming’en Zhang, Lei Yu, Guangyu Yan, Yan Chen, Xueliang Lin, Yiping Zhang, Zhuan Hong

**Affiliations:** 1 Engineering Technology Innovation Center for the Development and Utilization of Marine Living Resources, Third Institute of Oceanography, Ministry of Natural Resources, Xiamen 361005, China; 218527212@fzu.edu.cn (Y.Z.); chenhui@tio.org.cn (H.C.); 18558969140@163.com (M.Z.); ypzhang@tio.org.cn (Y.Z.); 2 Xiamen Ocean Vocational College, Xiamen 361100, China; yulei@xmoc.edu.cn (L.Y.); yanguangyu@xmoc.edu.cn (G.Y.); 3 Haijia Flour Milling Company Limited, China Oil & Foodstuffs Corporation, Xiamen 361026, China; 4 Fujian Key Laboratory of Island Monitoring and Ecological Development, Island Research Center, Ministry of Natural Resources, Pingtan 350400, China; 5 College of Advanced Manufacturing, Fuzhou University, Quanzhou 362200, China

**Keywords:** microcapsule, *Laminaria japonica* polysaccharide, gastrointestinal digestion, heat treatment, *Lactobacillus plantarum*, spray-drying

## Abstract

To improve probiotics’ survivability during gastrointestinal digestion and heat treatment, *Lactobacillus plantarum* was microencapsulated by spray-drying using *Laminaria japonica* polysaccharide/sodium caseinate/gelatin (LJP/SC/GE) composites. Thermogravimetry and differential scanning calorimetry results revealed that the denaturation of LJP/SC/GE microcapsules requires higher thermal energy than that of SC/GE microcapsules, and the addition of LJP may improve thermal stability. Zeta potential measurements indicated that, at low pH of the gastric fluid, the negatively charged LJP attracted the positively charged SC/GE, helping to maintain an intact microstructure without disintegration. The encapsulation efficiency of *L. plantarum*-loaded LJP/SC/GE microcapsules reached about 93.4%, and the survival rate was 46.9% in simulated gastric fluid (SGF) for 2 h and 96.0% in simulated intestinal fluid (SIF) for 2 h. In vitro release experiments showed that the LJP/SC/GE microcapsules could protect the viability of *L. plantarum* in SGF and release probiotics slowly in SIF. The cell survival of LJP/SC/GE microcapsules was significantly improved during the heat treatment compared to SC/GE microcapsules and free cells. LJP/SC/GE microcapsules can increase the survival of *L. plantarum* by maintaining the lactate dehydrogenase and Na^+^-K^+^-ATPase activity. Overall, this study demonstrates the great potential of LJP/SC/GE microcapsules to protect and deliver probiotics in food and pharmaceutical systems.

## 1. Introduction

Probiotics are living microorganisms that have health benefits for the host when administered in adequate amounts [1]. More and more strains have been proven to be probiotics and applied by humans, including *Lactobacillus* and *Bifidobacterium* [2]. *Lactobacillus plantarum* is a lactic acid bacterium. It has many probiotic functions, such as improving the gastrointestinal barrier function, preventing the overgrowth of pathogenic bacteria, anti-cardiovascular-disease activity, and immune regulation [3,4]. Probiotics are effective when they remain active and metabolically stable in the gastrointestinal tract and product [5]. However, *L. plantarum* has poor stability and is very sensitive to adverse environmental factors such as heat, stomach acid, digestive enzymes, and bile salts [3,6]. Adverse environments can reduce microbial activity by destroying cell membrane integrity, reducing the fluidity and permeability of cell membranes, and inactivating crucial metabolic enzymes [7]. Hence, most *L. plantarum* has difficulty reaching the gut to function as a probiotic. Microencapsulation is considered to be an effective technique to protect probiotics from adverse environmental conditions, ensuring that adequate amounts of probiotics enter the human body and are released in the gut [8].

Probiotic microencapsulation consists of entrapping probiotics in a tiny particle using natural or synthetic polymers as wall materials [9]. It can effectively prevent direct contact between the strain and an adverse external environment, alleviate cell damage, and reduce probiotics’ viability loss during processing, storage, and transport. In the preparation of probiotic microcapsules, the choice of the appropriate wall material has an essential impact on the survival rate, pH adaptation, temperature tolerance, and storage stability of the probiotics. Moreover, the stability of the microencapsulation matrix depends upon the strong intermolecular interactions of the materials [10]. Polysaccharides, proteins, and lipids are commonly used as wall materials for preparing probiotic microcapsules [11]. Among them, sodium caseinate (SC) is a natural pH-dependent protein with an isoelectric point of 4.6, which is biocompatible, edible, and biodegradable [12]. Gelatin (GE) is a protein obtained from the thermal denaturation of collagen. It is one of the most important natural polymer carrier materials. It has good emulsification properties, film formation, water solubility, high stabilizing activity, and a tendency to form a fine, dense network [13]. The combination of SC and GE can form a strong microcapsule membrane structure to effectively protect probiotics from external damage [14].

Seaweed polysaccharide has good emulsification, gelling, and film-forming properties and is an important wall material for microcapsules [15]. It can form three-dimensional gel network structures, substrates, or protective films, which protect and stabilize the embedded core material. *Laminaria japonica* is one of the most economically important seaweeds in China. *Laminaria japonica* polysaccharide (LJP) is a natural active substance extracted from *L. japonica*. Its main components are alginate, fucoidan, and laminarin [16]. LJP has been reported to improve intestinal flora disorders and obesity. It is beneficial to the human intestinal ecosystem and is considered to be a potential prebiotic [17]. The use of LJP in the wall material of probiotic microcapsules has rarely been reported. Moreover, the polysaccharide–protein matrix has shown great potential in delivering food-based bioactives [2]. This work used LJP, SC, and GE as composite wall materials to construct probiotic microcapsules. Taking *L. plantarum* as a representative probiotic strain, the protective effects of the composite microcapsules on probiotics during gastrointestinal digestion and heat treatments were evaluated. In addition, the morphology and structural properties of the microcapsules were analyzed. This study provides a new approach to the high-value utilization of *L. japonica* and the efficient construction of probiotic carriers, presenting promising applications in the fields of functional foods and pharmaceuticals. 

## 2. Results and Discussion

### 2.1. Morphology and Particle Size

**Figure 1:**
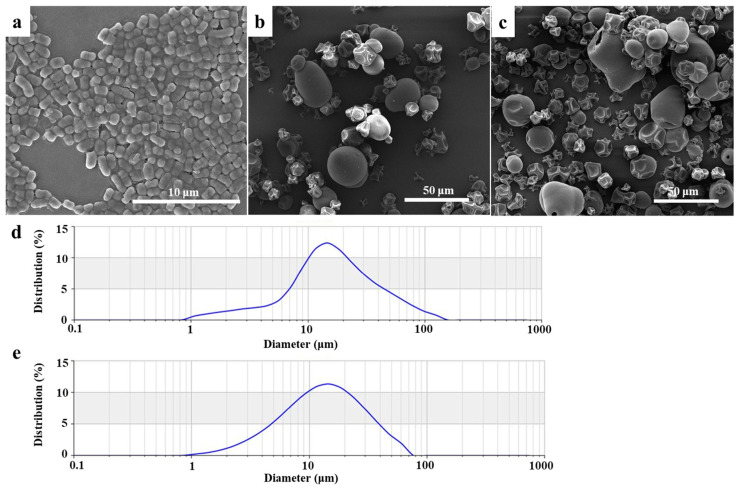
SEM images of (**a**) *L. plantarum*, (**b**) *L. plantarum*-loaded LJP/SC/GE microcapsules, and (**c**) *L. plantarum*-loaded SC/GE microcapsules. Particle size distribution of (**d**) *L. plantarum*-loaded LJP/SC/GE microcapsules and (**e**) *L. plantarum*-loaded SC/GE microcapsules.

The SEM images of *L. plantarum*, *L. plantarum*-loaded LJP/SC/GE microcapsules, and *L. plantarum*-loaded SC/GE microcapsules are presented in Figure 1. *L. plantarum* presents a short, rod-like shape with a length of 1~1.5 μm and a width of 0.6~0.8 μm (Figure 1a). *L. plantarum*-loaded LJP/SC/GE and SC/GE microcapsules exhibit a spheroidal shape with a wrinkled surface caused by the rapid evaporation of water during spray-drying. Compared to *L. plantarum*-loaded SC/GE microcapsules, *L. plantarum*-loaded LJP/SC/GE microcapsules have a smoother surface, without obvious holes and cracks, and do not easily leak bacteria. Although spray-dried microcapsules with concavities on the surface have been shown to be smaller in size than freeze-dried microcapsules, spray-drying can improve the mechanical strength and barrier performance of microcapsules and reduce the permeation of oxygen and digestive fluid [18]. No free *L. plantarum* cells were observed to emerge from the microcapsules, suggesting that the microcapsules could provide excellent encapsulation of *L. plantarum*. The microcapsule size was not homogeneous. The particle size of the *L. plantarum*-loaded LJP/SC/GE microcapsules ranged from 7 to 40 μm, and the typical particle size was about 14 μm. In contrast, the particle size of *L. plantarum*-loaded SC/GE microcapsules ranged from 4 to 30 μm, with a typical particle size of about 12 μm. The particle size distribution (Figure 1d,e) showed that the mean diameter of the *L. plantarum*-loaded LJP/SC/GE microcapsules and *L. plantarum*-loaded SC/GE microcapsules was about 14.5 and 12.4 µm, respectively, almost consistent with the SEM observation results.

### 2.2. FTIR Analysis

**Figure 2:**
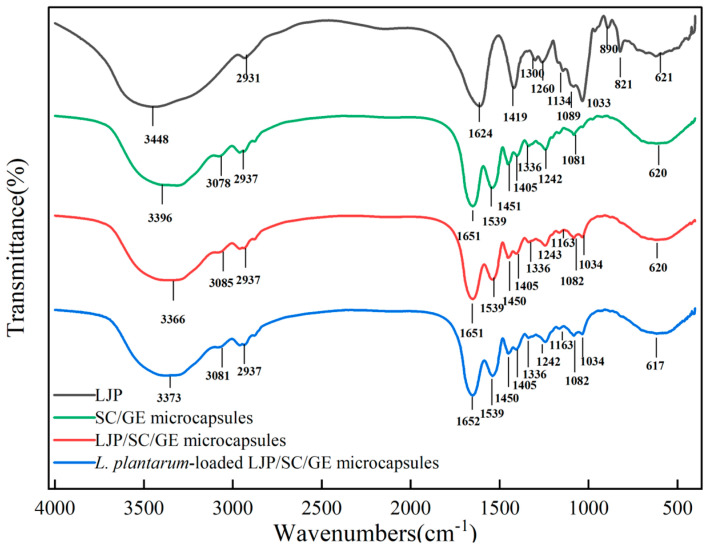
FTIR spectra of LJP, SC/GE microcapsules, LJP/SC/GE microcapsules, and *L. plantarum*-loaded LJP/SC/GE microcapsules.

In the FTIR spectrum of LJP (Figure 2), the peaks at 3448 cm^−1^ and 2931 cm^−1^ belonged to the stretching vibration of the O-H in the constituent sugar unit and C-H in the sugar ring, respectively [19]. The absorption bands at 1624 cm^−1^ and 1419 cm^−1^ were attributed to the asymmetrical (C=O) and symmetrical (C-O) stretching vibration of the carboxylate group. The peak at 1260 cm^−1^ was caused by the stretching vibration of O=S=O in the sulfate in the LJP. The absorption peaks at 1134 cm^−1^, 1089 cm^−1^, and 1033 cm^−1^ belonged to the asymmetric stretching vibration of glycosidic C-O-C bonds, indicating the existence of pyranose rings in LJP [20]. An absorption peak at 890 cm^−1^ represented the presence of β-pyranoses. The absorption band at 821 cm^−1^ suggested a C-O-S stretching vibration of sulfate groups on galactoyranose residues [19]. The sulfate content of the LJP was about 8%. The characteristic peaks at 1260 cm^−1^ and 821 cm^−1^ could confirm that the LJP was rich in polysaccharide sulfates. In addition, the position of the C-O-S peak in the 800~850 cm^−1^ region can predict the substitution position of the sulfate group. The peak at 821 cm^−1^ indicated that the substitution of the sulfate group occurred at the C-2 position [21]. The band at 621 cm^−1^ was probably due to the asymmetric ring vibration of uronic acid [22].

In the FTIR spectrum of the SC/GE microcapsules (Figure 2), the absorption band at 3396 cm^−1^ was caused by the amide A band, which was associated with stretching vibrations of O-H and N-H bonds. Amide B, associated with asymmetric tensile vibrations of =C-H and $–{\mathrm{NH}}_{3}^{+}$ was seen at 3078 cm^−1^ [23], while 2937 cm^−1^ was associated with the -CH_2_ stretching vibration. The absorption peak at 1651 cm^−1^ was mainly a C=O tensile vibration of the amide I band coupled with the C-N stretching. The amide II band at 1539 cm^−1^ was related to the symmetric stretching of N-C=O bonds. The bands at 1451 cm^−1^ and 1405 cm^−1^ were attributed to the C-H and -CH_3_ deformation vibrations. The peak at 1242 cm^−1^ was assigned to the combined peak between the C-N stretching vibration and N-H deformation of the amide III band. The band at 1081 cm^−1^ referred to the vibration of C-O [12]. 

When SC/GE was combined with LJP, the absorption bands of the amide A band and amide B band were significantly shifted to 3366 cm^−1^ and 3085 cm^−1^, respectively, which indicated that the amino groups in SC/GE formed strong hydrogen bonds with the hydroxyl groups in LJP [24]. Meanwhile, since the LJP contained about 10.2% uronic acid, there was also a strong hydrogen bond interaction between the large number of carbonyl groups in the LJP and the amino groups in the SC/GE. Due to the high temperatures of spray-drying, the amino groups of proteins might react with the carbonyl groups in LJP to form Schiff bases [25]. However, no characteristic peak of the Schiff base structure C=N was found in the range of 1690~1630 cm^−1^, indicating no covalent cross-linking between amino and carbonyl groups [26]. The characteristic peak of LJP at 1624 cm^−1^ vanished in LJP/SC/GE, suggesting an electrostatic attraction between the amine groups of the proteins and the carboxyl groups of the LJP [18]. A small absorption peak at 1034 cm^−1^ corresponded to the glycosidic C-O-C bond of the LJP. The main characteristic peaks in the FTIR spectrum of the *L. plantarum*-loaded LJP/SC/GE microcapsules were similar to those of empty LJP/SC/GE microcapsules. However, the distinct bands of amide and phosphate groups in the bacterial cell walls were not observed. This could be attributed to the high encapsulation efficiency of *L. plantarum* and the absence of bacteria on the surface of the microcapsules. The lack of significant shifts in the main characteristic peaks suggested that the formation of *L. plantarum*-loaded LJP/SC/GE microcapsules was based on hydrogen bonds, electrostatic interactions, and van der Waals forces rather than covalent bonds [27]. A similar result was obtained by Vaziri et al. (2018) [27] with alginate/pectin/gelatin biocomposites for the microencapsulation of *L. plantarum*.

### 2.3. Thermal Properties

**Figure 3:**
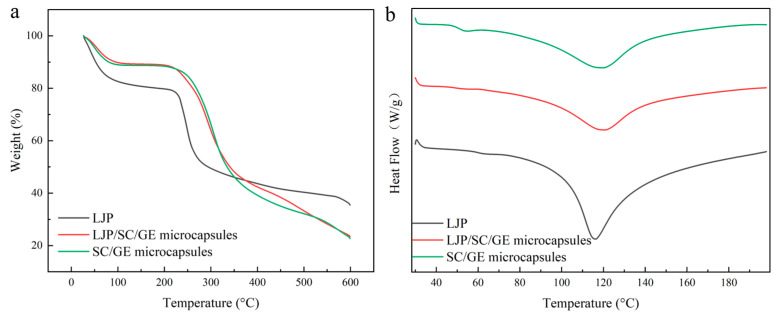
(**a**) TG and (**b**) DSC curves of LJP, SC/GE microcapsules, and LJP/SC/GE microcapsules.

The TG curves for LJP, SC/GE microcapsules, and LJP/SC/GE microcapsules are depicted in Figure 3a. It can be seen that there are almost two main weight loss stages: The first stage (30~200 °C) was due to the evaporation of free moisture [27]. The LJP exhibited more apparent weight loss than the microcapsule sample. The LJP lost 19.2% of its weight at 150.0 °C, while the SC/GE microcapsules and LJP/SC/GE microcapsules lost 11.3% and 10.8%, respectively. This indicates that the LJP was hydrophilic and had excellent water-holding capacity. The second stage was in the range of 200~400 °C. The weight loss at this stage was due to the decomposition of the biopolymers and the carbonates [28]. Polysaccharides are composed of functional groups of carboxylate or carboxylic acid. Thermal scission of the carboxylate groups and glycosidic bonds in LJP mainly occurred at 249.0 °C. When the LJP was combined with SC/GE, the thermal stability was improved. The degradation temperature of the LJP/SC/GE microcapsules was about 292.7 °C, which could be ascribed to the decomposition of protein and polysaccharide structures [29]. At 400.0 °C, the weight loss of the SC/GE microcapsules was about 61.0%, while that of the LJP/SC/GE microcapsules was 57.8%. Our observations are consistent with those of Duhoranimana et al. (2017), who reported that gelatin/carboxymethyl cellulose coacervates showed much gentler weight loss than gelatin up to 500 °C.

The DSC curves for LJP, SC/GE microcapsules, and LJP/SC/GE microcapsules are given in Figure 3b. The prominent endothermic peaks of the LJP, SC/GE microcapsules, and LJP/SC/GE microcapsules were observed at 115.9 °C, 119.2 °C, and 120.4 °C, respectively, possibly because of the denaturation of the wall material due to thermal melting at high temperatures, resulting in a shift from an ordered crystal structure to a disordered crystal structure [29,30]. The denaturation temperature range (ΔT_d_), peak denaturation temperature (T_d_), and denaturation enthalpy (ΔH_d_) were determined from the DSC heat flow curves with professional software (Universal Analysis version 4.5A, TA, USA), as shown in Table 1. The denaturation enthalpy (ΔH_d_) of the SC/GE microcapsules and LJP/SC/GE microcapsules was 172.5 J/g and 194.6 J/g, respectively. The results indicated that the denaturation of LJP/SC/GE microcapsules requires higher thermal energy than that of SC/GE microcapsules. The higher ΔH_d_ value of the LJP/SC/GE microcapsules revealed that the addition of LJP conferred stability to the protein matrix, and that the LJP/SC/GE composites were preferable as wall materials to encapsulate thermally sensitive core materials. The excellent thermal stability of the LJP/SC/GE microcapsules might be related to the monosaccharide composition of LJP, which was composed of 40.6% fucose, 27.3% galactose, 26.7% mannose, 2.0% arabinose, and 1.4% rhamnose [19]. The high contents of fucose and galactose were shown to be favorable for improved thermal stability [31]. These thermal performance results show good agreement with the findings reported by Duhoranimana et al. [29] and Timilsena et al. [32]. The enthalpy values of polysaccharide and protein composites were higher than the corresponding values of pure protein.

### 2.4. Zeta Potential

**Figure 4:**
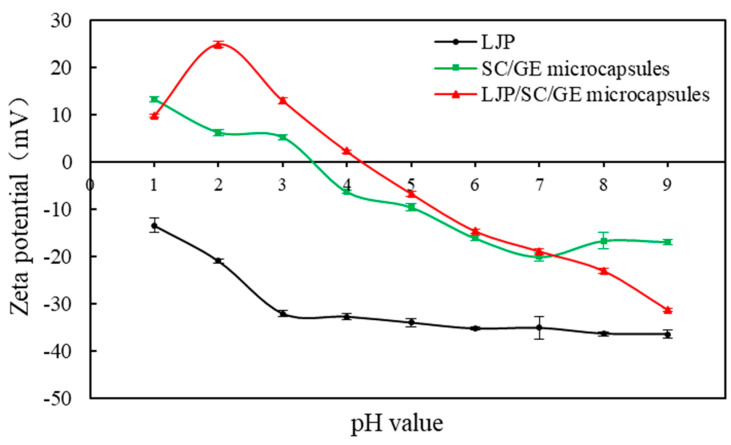
Zeta potential measurement of LJP, SC/GE microcapsules, and LJP/SC/GE microcapsules at different pH values (from 1 to 9). Data are presented as means ± SD, *n* = 3.

Figure 4 presents the zeta potential values of LJP, SC/GE microcapsules, and LJP/SC/GE microcapsules as a function of pH. It can be seen that, at pH 4.2, the surface charge of the LJP/SC/GE microcapsules was about zero. As the pH increased, the carboxylic groups in the LJP/SC/GE microcapsules became deprotonated and, thus, the negative charge gradually increased [33]. At pH 6.0, the zeta potential on the surface of the LJP/SC/GE microcapsules was −14.7 mV. A highly charged state above pH 6.0 might enhance the electrostatic repulsion between LJP, SC, and GE macromolecules and easily lead to the expansion of the microcapsule, thus allowing the desired release of the core material in SIF. For pH below 4.2, the surface of the LJP/SC/GE microcapsules was positively charged. The zeta potential value gradually increased with decreasing pH, reaching a maximum value of 24.8 mV at pH 2.0. However, the zeta potential decreased at pH values lower than 2.0, and the value dropped to about 9.7 mV at a low pH of 1.0. The electrical equivalence point pH of the SC/GE microcapsules occurred at about 3.5. The pI of SC was about 4.7, and that of GE was measured to be ~3.0 [14,34]. At SGF pH, both SC and GE macromolecules were positively charged, and the zeta potential on the surface of the SC/GE microcapsules was higher than that of the LJP/SC/GE microcapsules, indicating that the SC/GE microcapsules were more likely to cause structural loosening. As shown in Figure 4, LJP was negatively charged throughout the pH range, varying from −13.5 mV at pH 1.0 to −36.5 mV at pH 9.0, since LJP is a water-soluble anionic polysaccharide. Mao et al. and Lv et al. also found that carrageenan and gum arabic polysaccharides were negatively charged in a wide pH range [35,36]. At extremely low pH (pH 1~2), the absolute zeta potential value decreased because the dissociation of the carboxyl groups was suppressed [36]. When microcapsules were suspended in the SGF (pH 1.2), positively charged SC/GE interacted with negatively charged LJP molecules, which could enhance the electrostatic attraction between macromolecules and help to maintain an intact microstructure without disintegration.

### 2.5. Encapsulation Efficiency and Resistance to Simulated Gastrointestinal Fluid

**Figure 5:**
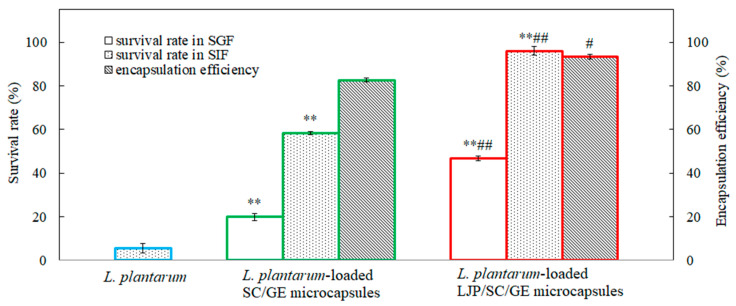
Encapsulation efficiency and survival rates of *L. plantarum*, *L. plantarum*-loaded SC/GE microcapsules, and *L. plantarum*-loaded LJP/SC/GE microcapsules in simulated gastrointestinal fluids (data are presented as means ± SD, *n* = 3; ** *p* < 0.01 versus free cells; # *p* < 0.05 and ## *p* < 0.01 versus SC/GE microcapsules).

Figure 5 shows the results of the encapsulation efficiency and survival rates of free and microencapsulated *L. plantarum* exposed to SGF and SIF with bile salt (0.3%). As shown in Figure 5, the encapsulation efficiency of the SC/GE microcapsules was about 82.6%, while that of the LJP/SC/GE microcapsules was about 93.4%. The LJP/SC/GE microcapsules exhibited much higher encapsulation efficiency than the SC/GE microcapsules (*p* < 0.05). This may have been due to the excellent film-forming performance of LJP, which can help form denser membranes and improve the encapsulation efficiency of microcapsules. Moreover, polysaccharides can lessen the microcapsules’ collapse and expedite cell adhesion with wall materials [37]. Thus, the combination of different polymers is favorable for improving the matrix’s structural issues [10]. Azam et al. [10] reported similar results, such as the addition of carrageenan, which reduced the pores of the alginate beads and improved the outer walls of the beads for higher encapsulation efficiency. In addition to the composition of the wall material, the polymer ratio also plays a vital role in the matrix stability and viability of microencapsulated cells. Excessive LJP would lead to particle aggregation, while too little LJP would be ineffective. The optimal microcapsule preparation conditions were 10 g/L SC, 20 g/L GE, and 5 g/L LJP, resulting in the highest encapsulation efficiency and survival rates.

The initial viable count for *L. plantarum* was about 10^9^ CFU/mL. After incubation in simulated gastrointestinal fluid for 2 h, the lowest survival rate was for free cells of *L. plantarum*, with a survival rate of ~0% and a cell density of approximately 1.1 log CFU/mL in SGF and 5.6% in SIF. The data showed that *L. plantarum* had extremely poor tolerance to both SGF and SIF. On the other side, higher survival rates were found in microencapsulated cells. The survival rate of *L. plantarum* in SC/GE microcapsules was about 19.9% and 58.2% in SGF and SIF, respectively, while *L. plantarum* in LJP/SC/GE microcapsules survived significantly better (*p* < 0.01). When exposed to SGF (pH 1.2) for 2 h, the viable count of the LJP/SC/GE microcapsules decreased by 0.3 log CFU/mL, with a survival rate of 46.9%. Furthermore, the viable count of the LJP/SC/GE microcapsules remained almost constant, with a survival rate of approximately 96.0% when treated in SIF for 2 h. 

The experimental results were consistent with the zeta potential measurements. At SGF pH, both SC and GE carried positive charges. However, abundant cationic counterions (from SC) could reduce the repulsive interactions between the SC and GE. The poor electrostatic linkages between SC and GE may allow the microcapsules to expand and absorb water, buffering the penetration of acidic compounds [34]. LJP, as a negatively charged polysaccharide, further enhances such a buffer effect [38]. *L. plantarum* cells were also positively charged at SGF pH, which made the cells strongly attracted to LJP. This interaction might promote cell adhesion in microcapsules and improve bacterial tolerance to SGF [35]. Thus, the combination of LJP, SC, and GE might increase the adhesion and interaction between the polysaccharide and the protein matrix and between the bacteria and the wall material, forming a more rigid structure. It was concluded that the LJP/SC/GE microcapsules significantly protected *L. plantarum* from gastric acid, bile salt, and digestive enzyme injury.

**Figure 6:**
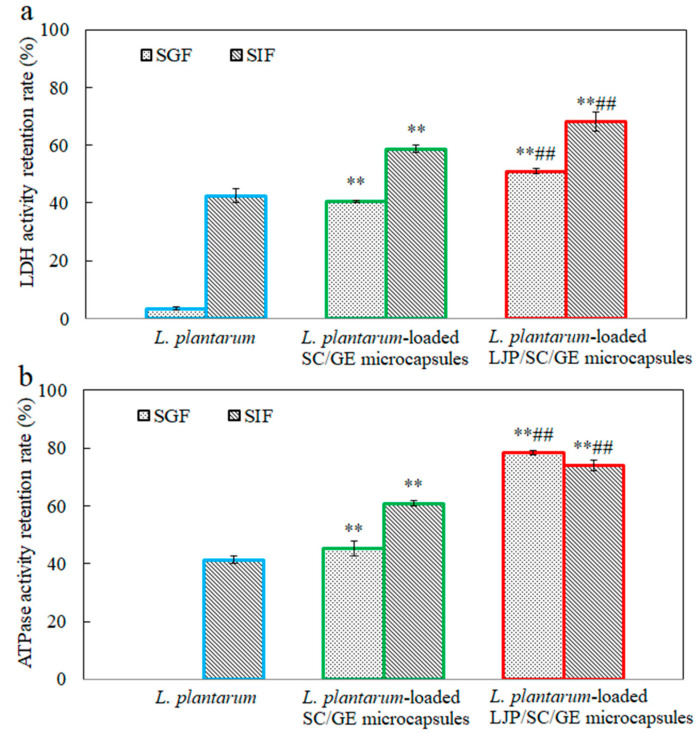
Retention rates of (**a**) LDH and (**b**) Na^+^-K^+^-ATPase activity in *L. plantarum*, *L. plantarum*-loaded SC/GE microcapsules, and *L. plantarum*-loaded LJP/SC/GE microcapsules after simulated gastrointestinal fluid treatment (data are presented as means ± SD, *n* = 3; ** *p* < 0.01 versus free cells; ## *p* < 0.01 versus SC/GE microcapsules).

Figure 6 shows the effects of SGF and SIF on the LDH and Na^+^-K^+^-ATPase activity of free and microencapsulated *L. plantarum*. LDH and ATPase are critical enzymes in *L. plantarum*. LDH catalyzes the conversion of pyruvate to lactate. Na^+^-K^+^-ATPase is an integral membrane enzyme necessary to maintain normal cell function and is a sensitive monitor of environmental changes. The cellular LDH and Na^+^-K^+^-ATPase activity can reflect the viability and metabolic activity of probiotics [39,40]. It has been observed that both LDH and Na^+^-K^+^-ATPase activity can be impaired by digestive fluid treatment. After incubation in SGF for 2 h, the retention rates of LDH and Na^+^-K^+^-ATPase activity in free cells decreased to only 3.4% and 0%, respectively. Enzyme activity was less affected by SIF than by SGF. When exposed to SIF for 2 h, the retention rates of LDH and Na^+^-K^+^-ATPase activity in free cells were 42.4% and 41.3%, respectively. The retention rates of LDH and Na^+^-K^+^-ATPase activity in microencapsulated cells were much higher than those in free cells (*p* < 0.01). Compared with the SC/GE microcapsules, the LJP/SC/GE microcapsules could more significantly maintain the critical enzyme activity (*p* < 0.01). This observation was in accordance with the changes in the cell survival of free and microencapsulated *L. plantarum* in simulated gastrointestinal fluid (Figure 5). These data suggest that LJP/SC/GE microcapsules can increase the survival of *L. plantarum* by maintaining the LDH and Na^+^-K^+^-ATPase activity.

### 2.6. In Vitro Release

**Figure 7:**
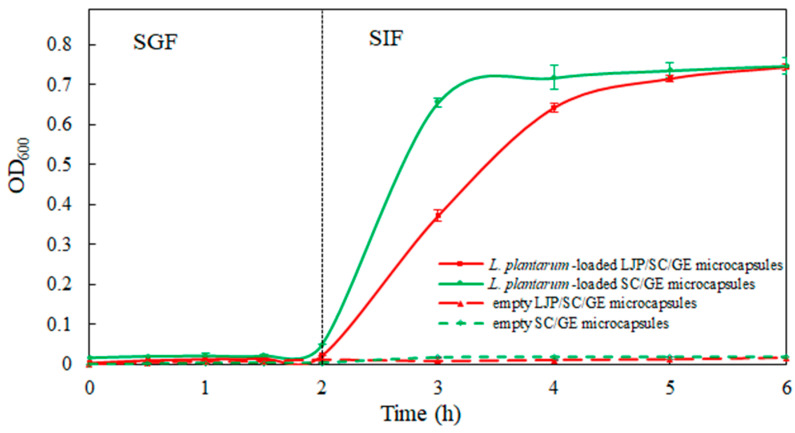
In vitro release profiles of empty LJP/SC/GE microcapsules, empty SC/GE microcapsules, *L. plantarum*-loaded SC/GE microcapsules, and *L. plantarum*-loaded LJP/SC/GE microcapsules in SGF and SIF (data are presented as means ± SD, *n* = 3).

Empty microcapsules and *L. plantarum*-loaded microcapsules were incubated in SGF for 2 h and in SIF for 4 h to assess the release performance of the microcapsules. The in vitro release profiles are shown in Figure 7. As can be seen, the release media of empty LJP/SC/GE microcapsules and empty SC/GE microcapsules had a low absorbance over the incubation time in SGF and SIF, which could not affect the detection of *L. plantarum* release. During the incubation in SGF for 2 h, cell release from *L. plantarum*-loaded microcapsules was minor, with an OD_600_ of 0.050 for SC/GE microcapsules and an OD_600_ of 0.022 for LJP/SC/GE microcapsules, indicating that the microcapsules did not disintegrate in SGF. As a result, encapsulated *L. plantanum* cells were resistant to the gastric acid environment, and the LJP/SC/GE microcapsules were more stable in SGF than the SC/GE microcapsules. The strong electrostatic interaction between the LJP and the proteins was the major driving force for maintaining the structural integrity of the microcapsules, inhibiting pepsin and H^+^ access [18,35]. It was observed that the cell release rate from the microcapsules was accelerated when exposed to SIF. The *L. plantarum*-loaded SC/GE microcapsules began disintegrating in SIF, and the OD_600_ value increased to 0.656 within 1 h. In contrast, the LJP/SC/GE microcapsules showed a slow release of *L. plantarum*. The OD_600_ gradually increased to 0.372 within 1 h, followed by full cell release at approximately 3 h of incubation in SIF, with an OD_600_ of 0.715. The gelling properties of polysaccharides may be the main factor leading to slow release [10]. Adding LJP improved the structure and buffered the SIF’s diffusion process into the microcapsules. Therefore, LJP/SC/GE microcapsules offer better protection for probiotics, with more controlled and sustained release than SC/GE microcapsules. Similar results were obtained by Azam et al. [10]. The alginate beads presented burst release of *L. brevis* LBR1 in SIF, while adding carrageenan to the alginate beads showed slower release and better protection for *L. brevis* LBR1.

### 2.7. Resistance to Heat Treatments

**Figure 8:**
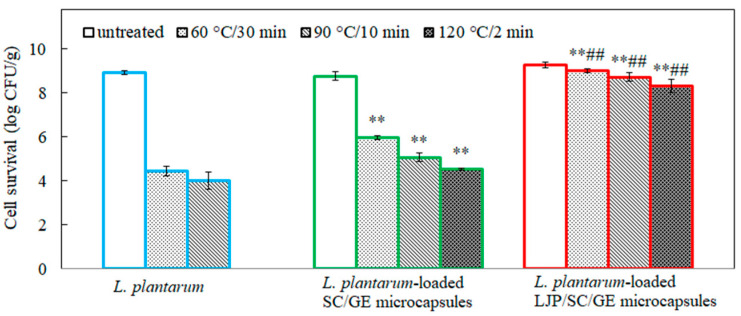
Survival of *L. plantarum*, *L. plantarum*-loaded SC/GE microcapsules, and *L. plantarum*-loaded LJP/SC/GE microcapsules at different temperatures (60 °C, 90 °C, and 120 °C). Data are presented as means ± SD, *n* = 3; ** *p* < 0.01 versus free cells; ## *p* < 0.01 versus SC/GE microcapsules.

The effect of temperature (60 °C, 90 °C, and 120 °C) on the survival of *L. plantarum* is shown in Figure 8. *L. plantarum* cells were highly sensitive to heat treatment. After heat treatments of 60 °C for 30 min and 90 °C for 10 min, the survival of *L. plantarum* cells decreased from 8.92 log CFU/g to 4.45 and 4.02 log CFU/g, respectively. At 120 °C, free cells were totally inactivated within 2 min. In contrast, the microencapsulated *L. plantarum* showed better heat resistance. At 60 °C for 30 min, a decrease of 2.79 log CFU/g was obtained for the SC/GE microcapsules, while only a reduction of 0.24 log CFU/g was observed for the LJP/SC/GE microcapsules. After heat treatment of 90 °C for 10 min, the *L. plantarum*-loaded SC/GE microcapsules showed a reduction of 3.70 log CFU/g, but the corresponding value was 0.53 log CFU/g for *L. plantarum*-loaded LJP/SC/GE microcapsules. Moreover, the cell survival of LJP/SC/GE microcapsules exposed to 120 °C for 2 min was significantly higher than that of SC/GE microcapsules (*p* < 0.01). In addition, after the spray-drying process, the survival rate of *L. plantarum* cells was 0%. In contrast, the survival rates of *L. plantarum*-loaded SC/GE and LJP/SC/GE microcapsules were about 43% and 55%, respectively. Overall, these results demonstrated that *L. plantarum* was found to have the highest cell survival in LJP/SC/GE microcapsules. The above TG and DSC results also indicate better thermal stability of the LJP/SC/GE microcapsules. The heat tolerance of probiotics was closely related to the wall material formulation. The presence of substrates, especially the addition of LJP, could significantly increase the thermotolerance of encapsulated probiotics. In addition to the monosaccharide composition of LJP, this might also be attributable to the polysaccharide–protein matrix. There was good adhesion between the LJP and the proteins. The FTIR analysis showed that hydrogen bonds and electrostatic attraction occurred between LJP and SC/GE, leading to strong non-covalent interactions. This strong interaction can increase the mechanical strength of the microcapsule membrane and make the microstructure more compact and dense, which could effectively slow down or isolate the effect of heating on probiotics [1,41]. Since there was no such strong interaction in the pure protein matrix, its thermal protection effect was inferior to that of the polysaccharide–protein matrix. A similar result was published by Guo et al.; in their thermotolerance test, the whey protein isolate–dextran conjugate enhanced the survival of encapsulated cells, and the viable count of encapsulated *L. plantarum* decreased from about 8.3 log CFU/mL to 3.2 log CFU/mL after heat treatment at 85 °C for 2 min. In contrast, free cells were inactivated within 1 min [42].

**Figure 9:**
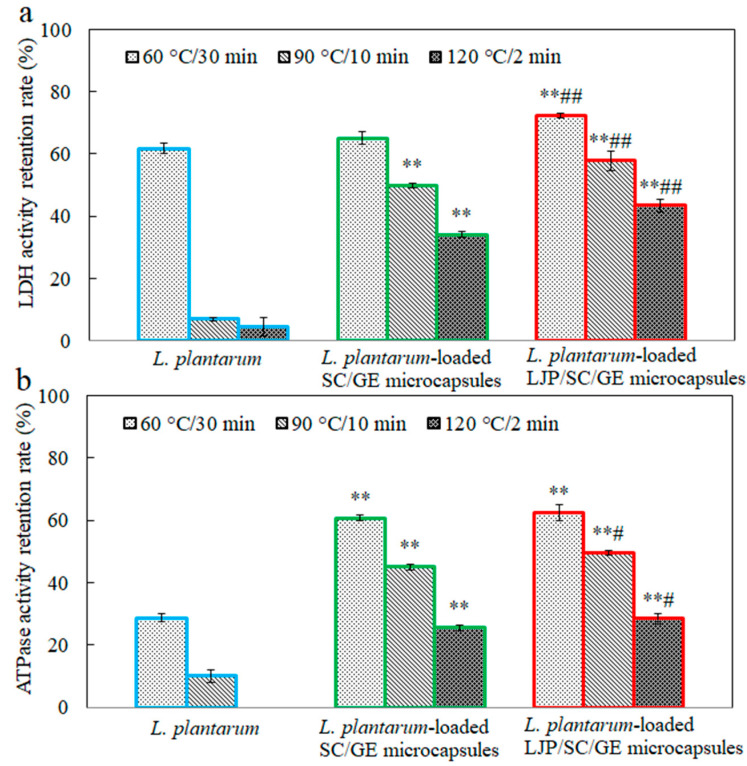
Retention rates of (**a**) LDH and (**b**) Na^+^-K^+^-ATPase activity in *L. plantarum*, *L. plantarum*-loaded SC/GE microcapsules, and *L. plantarum*-loaded LJP/SC/GE microcapsules after heat treatments (data are presented as means ± SD, *n* = 3; ** *p* < 0.01 versus free cells; # *p* < 0.05 and ## *p* < 0.01 versus SC/GE microcapsules).

Figure 9 depicts the effects of temperature (60 °C, 90 °C, and 120 °C) on the LDH and Na^+^-K^+^-ATPase activity in free and microencapsulated *L. plantarum*. After heat treatments, the LDH and Na^+^-K^+^-ATPase activity in free cells was significantly lower (*p* < 0.01) than in untreated cells. Especially at 120 °C for 2 min, the Na^+^-K^+^-ATPase activity decreased from 8.7 U/g to 0 U/g, with a retention rate of 0%. It can be seen that the heat treatment significantly damages the LDH and Na^+^-K^+^-ATPase activity, which severely affects the viability of the probiotics. Zhen et al. also reported that heat treatments affected the LDH and Na^+^-K^+^-ATPase activity significantly more than hexokinase, pyruvate kinase, phosphofructokinase, and Ca^2+^-ATPase [39]. The microencapsulated cells maintained higher LDH and Na^+^-K^+^-ATPase activity than the free cells, and a significant increase in the retention rate of enzyme activity was observed with the addition of LJP. This result was consistent with the changes in the cell survival of free and microencapsulated *L. plantarum* under the same heat treatments (Figure 8). These data suggest that LJP might act as a thermoprotectant, reducing cell damage when heated. Studies demonstrated that the thermoprotective action of some saccharides was to maintain the unique liquid crystalline configuration of the membrane lipid bilayer by replacing hydrogen-bonded water in the membrane bilayer during the drying process [43]. Xylooligosaccharide and soy polysaccharide could protect cellular LDH activity, possibly due to the hydrogen bonds between proteins and sugars [7]. LJP may protect the membrane via a similar mechanism. The polysaccharide–protein matrix might also enhance the interfacial properties and effective microencapsulation of probiotic bacteria [37]. Thus, the LJP/SC/GE carrier matrix acts as a physical shield for the probiotics and may alleviate the thermal and osmotic stresses on the bacteria during heating.

## 3. Materials and Methods

### 3.1. Materials

*L. japonica* was purchased from Fujian Yuanyang Algae Industry Co., Ltd. (Zhangzhou, China). The *L. plantarum* MCCC 1K05759 (DSM 20174) strain was provided by the Marine Culture Collection of China (Xiamen, China). Man–Rogosa–Sharpe (MRS) broth and agar were purchased from Solarbio Technology Co., Ltd. (Beijing, China). SC was purchased from Henan Qianbo Chemical Products Co., Ltd (Zhengzhou, China). GE was purchased from Xilong Scientific Co., Ltd (Shantou, China). Pepsin and trypsin were purchased from Macklin Biochemical Technology Co., Ltd. (Shanghai, China). Bile salt was purchased from Sinopharm Chemical Reagent Co., Ltd. (Shanghai, China). Na^+^ K^+^-ATPase activity assay kits and lacate dehydrogenase (LDH) activity assay kits were purchased from Solarbio Technology Co., Ltd (Beijing, China).

Preparation of simulated gastric fluid (SGF) was as follows [43]: 1.0 g of pepsin was dissolved in 80 mL of ultrapure water. The pH was adjusted to 1.2 with 10% hydrochloric acid solution. Then, the solution was diluted to 100 mL and filtered with a 0.22 μm sterile filter membrane for use.

Preparation of simulated intestinal fluid (SIF) was as follows [44,45]: 0.1 g of trypsin and 0.3 g of bile salt were dissolved in 100 mL of 0.2 mol/L phosphate buffer with pH 6.8, and then filtered with 0.22 μm sterile filter membrane for use.

### 3.2. Preparation of LJP

The preparation method of LJP was as described in our previous report [19]. In detail, *L. japonica* was cut into strips and washed clearly to remove the salt, followed by two rounds of delipidation. *L. japonica* was then dried and made into a 24-mesh powder. About 1 kg of *L. japonica* powder was extracted with 50 L of water at 90 °C for 3 h. The extraction process was repeated twice. Finally, the extracted LJP was collected, concentrated, and spray-dried. The yield of LJP was about 15%. It contained carbohydrates (31.30%), protein (4.51%), sulfate (13.70%), and water (9.53%), while the ash content was 19.65% [19].

### 3.3. Bacterial Cultivation

*L. plantarum* was incubated in MRS broth at 37 °C for 14 h in a shaking incubator at 120 rpm. Then, *L. plantarum* was subcultured (37 °C, 14 h) twice in MRS broth for activation and adaptation. The cells were centrifuged at 8000 rpm in a high-speed refrigerated centrifuge (CR22N, Hitachi, Tokyo, Japan) for 10 min to remove the medium. Fresh cells were subsequently subjected to microencapsulation. All of the media and glassware used in this procedure were sterilized at 121 °C for 20 min.

### 3.4. Microencapsulation of L. plantarum

Fresh *L. plantarum* cells were mixed with 10 g/L of SC solution. The cells were suspended to a final viable concentration of approximately 10^9^ CFU/mL. Then, 20 g/L of GE solution and 5 g/L of LJP solution were gently added, and the mixture was stirred for about 30 min. Finally, the mixture was spray-dried by a laboratory-scale spray-dryer (SY-6000 spray-dryer, Shiyuan, Shanghai, China) operating at a constant inlet temperature of 120 °C and outlet temperature of 80 °C. In the spray-drying process, the mixture was continuously stirred at room temperature and was pumped into the main chamber with a feed flow of 15 mL/min. After finishing the spray-drying, the microcapsule samples were obtained from the cyclone bottom and stored in sealed sterile vials at 4 °C.

### 3.5. Microcapsule Characterization

#### 3.5.1. Morphology and Particle size

The morphology was detected by scanning electron microscope (SEM, Hitachi Regulus 8100, Hitachi Ltd., Tokyo, Japan). The *L. plantarum*-loaded microcapsules were placed on a piece of conductive adhesive, and the excess powder was removed and coated with gold by vacuum sputtering for SEM observation. Particle size distribution was detected by a particle size analyzer (LS-POP (9), Zhuhai Omec Instrument Co., Ltd., Zhuhai, China). About 60 mL of 1~2 mg/mL *L. plantarum*-loaded microcapsule solution was added to the sample cell, and the shading ratio was set at 10~15% for testing.

#### 3.5.2. Infrared

The Fourier-transform infrared (FTIR) spectroscopy of the microcapsules was performed using a Fourier-transform infrared spectrometer (Thermofisher Niolet iN10, Thermo Fisher Scientific, Waltham, MA, USA). The samples were thoroughly mixed with KBr and then pressed into tablets for FTIR detection in the 4000~400 cm^−1^ range.

#### 3.5.3. Thermal Properties

The thermal properties of the samples were measured by a thermogravimetric analyzer (Discovery TGA 550, TA Instruments, New Castle, DE, USA) and differential scanning calorimeter (DSC 25, TA Instruments, New Castle, DE, USA). For thermogravimetric (TG) analysis, 3~10 mg of sample was placed onto the sample tray and heated from 25 °C to 600 °C at 10 °C/min in a nitrogen environment. A plot of the weight versus temperature was obtained. For differential scanning calorimetry (DSC) detection, about 3~4 mg of the sample was weighed in an aluminum pan. Nitrogen was used as a carrier gas. The samples were heated from 30 °C to 200 °C at a heating rate of 10 °C/min. The denaturation temperature range (ΔT_d_), peak denaturation temperature (T_d_), and enthalpy of denaturation (ΔH_d_) were calculated using the associated software (Universal Analysis version 4.5A, TA, USA).

#### 3.5.4. Zeta Potential Determination

Zeta potential was determined using a zeta potential analyzer (Malvern Zetasizer Nano ZS90, Malvern Instruments, Worcestershire, UK). The sample was dispersed in Britton–Robinson buffer solutions of different pH values at a 0.5 mg/mL concentration to measure the zeta potential.

#### 3.5.5. Encapsulation Efficiency

Next, 0.1 g of *L. plantarum*-loaded microcapsules was disintegrated in 4 mL of filter-sterilized phosphate buffer (0.1 mol/L, pH 7.0) with 0.2 mol/L NaHCO_3_ for the determination of the total viable count of *L. plantarum* in the microcapsules. Another 0.1 g of *L. plantarum*-loaded microcapsules was suspended in 4 mL of filter-sterilized buffer solution (pH 4.0) at 37 °C for 45 min under 100 rpm, and the supernatant was collected to determine the viable count of non-microencapsulated *L. plantarum*. *L. plantarum*’s viability was quantified by using the plate counting method. Viable cells were counted in terms of colony-forming units (CFU) after incubation in MRS agar at 37 °C for 48 h. The encapsulation efficiency was calculated based on the following equation [14,46]:

encapsulation efficiency (%) = (N − N_0_)/N × 100 (1)

where N is the total viable count of *L. plantarum*-loaded microcapsules, and N_0_ is the viable count of non-microencapsulated *L. plantarum*.

#### 3.5.6. Survival Rate in Simulated Gastrointestinal Fluid

Next, 0.1 g of *L. plantarum*-loaded microcapsules was added to 5.0 mL of pre-warmed SGF or SIF and incubated for 2 h at 37 °C under 100 rpm. Then, the sample was centrifuged at 8000 rpm for 10 min to separate the cells from the SGF or SIF, and then washed twice with sterile water. The survival rate was calculated based on the following equation:

survival rate (%) = N_1_/N × 100 (2)

where N is the initial viable count of *L. plantarum* in microcapsules, and N_1_ is the viable count of *L. plantarum* in microcapsules treated with SGF or SIF.

#### 3.5.7. Determination of Metabolic Enzyme Activity 

*L. plantarum*-loaded microcapsules were dispersed with ultrapure water and centrifuged at 8000 rpm for 10 min. The supernatant was discarded, bacteria were collected, and the cells were disrupted at 0 °C using an ultrasonic cell disruptor (power 40%, ultrasonic for 3 s, interval of 10 s, repeated 40 times). The metabolic enzyme activity of *L. plantarum* was determined using Na^+^ K^+^-ATPase and LDH activity assay kits. The retention rate of enzyme activity was calculated based on the following equation:

retention rate (%) = A_1_/A_0_ × 100 (3)

where A_0_ is the initial enzyme activity of *L. plantarum*, and A_1_ is the enzyme activity of *L. plantarum* treated with simulated digestive fluid or heat.

#### 3.5.8. In Vitro Release Studies

Next, 0.1 g of *L. plantarum*-loaded microcapsules was added to 20.0 mL of SGF and incubated at 37 °C for 2 h under orbital shaking at 100 rpm. After the SGF treatment, the sample was subsequently transferred to the SIF. The sample was then incubated in SIF at 37 °C for 4 h at 100 rpm. Empty microcapsules were used as a control. Then, 0.2 mL was taken out of the sample at 30-min intervals and replaced with fresh medium simultaneously. The OD_600_ of each sample was assayed in triplicate using an ultraviolet/visible microplate spectrophotometer (Multiskan SkyHigh, Thermo Scientific, Waltham, MA, USA).

#### 3.5.9. Heat Treatments 

Next, 0.1 g of *L. plantarum*-loaded microcapsules was placed in a sterile penicillin vial at 60 °C, 90 °C, and 120 °C for 30 min, 10 min, and 2 min, respectively. Free cells were used as a control. 

### 3.6. Statistical Analysis

All of the experiments were carried out in triplicate. The original data were averaged and processed using Excel 2013 or SPSS 16.0. An analysis of variance (ANOVA) was used to evaluate the significance of differences between the mean values. A *p*-value < 0.05 indicates statistically significant differences.

## 4. Conclusions

The severe conditions of gastrointestinal digestion and heat treatment can result in the inactivation of *L. plantarum*. Microencapsulation of *L. plantarum* with LJP/SC/GE composites can provide excellent protection to the bacteria during gastrointestinal digestion and heat treatments. Compared to SC/GE microcapsules, LJP/SC/GE microcapsules exhibited much better encapsulation efficiency and higher cell survival when exposed to SGF and SIF with bile salt. In particular, the addition of LJP gave the microcapsules a good slow-release effect in SIF. During heat treatments, the survival of *L. plantarum* in the LJP/SC/GE microcapsules was significantly enhanced, and cell damage was also reduced. The protective effect was attributed primarily to the polysaccharide–protein matrix, which effectively slowed or isolated the transfer of heat to the probiotics. Moreover, the addition of LJP was preferred to improve the thermal stability, due to its high fucose and galactose content. The present study suggests that LJP/SC/GE composite microcapsules with excellent stress resistance are ideal carriers for bacteria and have great prospects for food and pharmaceutical applications. 

## Figures and Tables

**Table 1:** Denaturation temperature range (ΔT_d_), peak denaturation temperature (T_d_), and denaturation enthalpy (ΔH_d_) of LJP, SC/GE microcapsules, and LJP/SC/GE microcapsules.

Samples	ΔT_d_ (°C)	T_d_ (°C)	ΔH_d_ (J/g)
LJP	102.5~142.2	115.9	284.3
SC/GE microcapsules	84.5~137.5	119.2	172.5
LJP/SC/GE microcapsules	89.2~138.3	120.4	194.6

## Data Availability

The original data presented in the study are included in the article; further inquiries can be directed to the corresponding author.
